# Severe acute respiratory syndrome coronavirus-2 shedding in exhaled material: a systematic review

**DOI:** 10.1017/S0950268825100174

**Published:** 2025-06-20

**Authors:** A. M. Hasanthi Abeykoon, Madeleine Wilson, Kanta Subbarao, Nicholas Geard, Cameron Zachreson, Sheena Geraldine Sullivan

**Affiliations:** 1 Department of Infectious Diseases, Melbourne Medical School, University of Melbourne, The Peter Doherty Institute for Infection and Immunity, Melbourne, VIC, Australia; 2 WHO Collaborating Centre for Reference and Research on Influenza, Royal Melbourne Hospital, Peter Doherty Institute for Infection and Immunity, Melbourne, VIC, Australia; 3 Department of Microbiology and Immunology, University of Melbourne, Peter Doherty Institute for Infection and Immunity, Melbourne, VIC, Australia; 4 Department of Microbiology and Immunology, Laval University, Quebec City, QC, Canada; 5 School of Computing and Information Systems, The University of Melbourne, Melbourne, VIC, Australia; 6 School of Clinical Sciences, Monash University, Melbourne, VIC, Australia

**Keywords:** Severe acute respiratory syndrome coronavirus-2, COVID-193, Systematic review, Exhaled breath, Replication competent

## Abstract

This systematic review synthesized evidence on the viral load of severe acute respiratory syndrome coronavirus-2 (SARS-CoV-2) shedding in exhaled material to understand how the exhaled SARS-CoV-2 viral load of infected individuals varies with days since exposure. Medline, Scopus, and Web of Science databases were searched using a combination of search terms to identify articles that tested exhaled material from SARS-CoV-2 infected patients. Records were systematically screened and assessed for eligibility, following which reference lists of eligible articles were hand-searched to identify further relevant studies. Data extraction and quality assessment of individual studies were conducted prior to synthesizing the evidence. Forty-five articles that sampled exhaled breath, exhaled breath condensate, face masks, and cough samples were reviewed. The variation in the SARS-CoV-2 viral load in these materials was considerable with the detection of viral RNA shed during breathing as far as 43 days after symptom onset. The replication-competent virus was present in all four sample types, with the majority isolated during the first week of symptoms onset. Variations in the sample types and testing protocols precluded meta-analysis. High heterogeneity in exhaled SARS-CoV-2 viral load is likely due to host and viral factors as well as variations in sampling and diagnostic methodologies. Evidence on SARS-CoV-2 shedding in exhaled material is scarce and more controlled fundamental studies are needed to assess this important route of viral shedding.

## Introduction

The rapid spread of severe acute respiratory syndrome coronavirus-2 (SARS-CoV-2) caused unprecedented strain on social, economic, and healthcare systems worldwide. Even with the use of new vaccines and antiviral treatments, SARS-CoV-2 remains difficult to control. One of the major contributing factors to this challenge is the nature of its transmission [1]. Early in the pandemic direct transmission by respiratory droplets among close contacts was assumed to be the only mode of transmission [3]. We now know that respiratory droplets, aerosols, and fomites can all transmit SARS-CoV-2 [2], with aerosols being the main mechanism of transmission [5–7].

Although droplet transmission and aerosol transmission are different parts of the same mechanism, the latter is the most effective mode of transmission as it creates small (<0.2 μm to >20 μm [4]) infectious particles capable of remaining airborne longer [2], thereby increasing the potential for infecting a susceptible host [8, 9]. Aerosols in hospitals both proximate and distant from SARS-CoV-2-positive patients can be contaminated with SARS-CoV-2 RNA or live virus [10]. The amount and duration of viral shedding by the infected individual is an important determinant of viral load in indoor air. Estimates of the latter are used to inform isolation and quarantine policies, to determine indoor crowding capacity and airborne transmission potential, for planning to prevent nosocomial outbreaks, and for retrospective outbreak analysis. However, precise measurement of aerosol shedding is challenging.

Since aerosol transmission is the dominant mode of COVID-19 spread, understanding viral load in the exhaled material of infected hosts is important. Here, the term “exhaled material” is used to denote a broad range of sample types including exhaled breath, exhaled breath condensate, condensed or absorbed material deposited on the inside of face masks by the infected person when wearing it, and coughed-up material. The types of samples that can be used to measure viral load, other than those mentioned above include respiratory swabs and respiratory fluids out of which nasopharyngeal and oropharyngeal swabs are the most common. Testing exhaled material is advantageous over swab sampling due to the non-invasive nature of the technique. However, it also has drawbacks such as requiring longer sampling times and the viral load may be several orders of magnitude lower compared to respiratory swabs and therefore exhaled material may have low sensitivity for the detection of the virus [11].

In order to contribute to airborne transmission, the virus needs to be exhaled, and hence it is important to determine the viral load actually entering the environment and directly contributing to exposure and transmission risk. Although the evidence on SARS-CoV-2 shedding as measured by respiratory swabs has been reviewed [12–15], to the best of our knowledge, the evidence that comes solely from exhaled material has not been synthesized. The current systematic review aimed to understand SARS-CoV-2 viral load shedding in exhaled material. We focus on how exhaled SARS-CoV-2 viral load varies in terms of days since exposure, and how long replication-competent virus is shed over the course of infection.

## Methods

### Search strategy

The comprehensive search strategy, shown in Table 1, included search terms relating to the key concepts of detecting SARS-CoV-2 and the type of matrix involved, i.e. exhaled material. This search strategy was used to identify articles published up to 16 November 2023 in Medline, Scopus, and Web of Science (all databases). Once eligible articles were identified, their reference lists were searched manually to identify further relevant articles. The review was restricted to original articles published in the English language. Both full-length articles and published abstracts were considered.

**Table 1.** Comprehensive search strategy and number of results from each database

Database	Search strategy	# Results
Ovid Medline	1. COVID–19/ 2. SARS-CoV–2/ 3. Severe acute respiratory syndrome coronavirus–2.mp. 4. Rovel coronavirus 2019.mp. 5. 1 or 2 or 3 or 4 6. Viability.mp. 7. Viral Load/ 8. Replicat*.mp. 9. Infectious virus.mp. 10. Viable.mp. 11. Viral culture.mp. 12. 6 or 7 or 8 or 9 or 10 or 11 13. air*.mp. 14. exhaled.mp. 15. Aerosol.mp. 16. 13 or 14 or 15 17. 5 and 12 and 16	777
Scopus	(TITLE (‘covid–19’ OR ‘sars-cov–2’ OR “‘severe acute respiratory syndrome coronavirus–2” OR “novel coronavirus 2019”) AND TITLE-ABS-KEY (viability OR “viral load” OR replicat* OR’ “infectious virus” OR viable OR “viral culture”) AND TITLE-ABS-KEY (air* OR exhaled OR aerosol))	599
Web of Science (all databases)	covid–19 OR sars-cov–2 OR “’severe acute respiratory syndrome coronavirus–2” OR “novel coronavirus 2019” (Title) AND viability OR “viral load” OR replicat* OR ’ “infectious virus” OR viable OR “viral culture” (Topic) AND air* OR exhaled OR aerosol (Topic)	1,370

### Article selection

All resulting articles from three electronic databases were exported to the reference management software EndNote 20.3. Duplicate records were systematically removed, and the remaining articles were initially screened based solely on the title and the abstract. Those articles that were clearly irrelevant were not considered further, while the remaining were retained for full-text review. During the full-text assessment, articles describing studies according to the following criteria were included: confirmed COVID-19 patients of any age, any duration of infection, and any symptomatic stage; sampled exhaled material, including exhaled breath, exhaled breath condensate, material deposited on face masks and coughed-up material and tested for either SARS-CoV-2 RNA or live virus or both. Exhaled material is defined as liquid or air, or a mixture of both, emitted from an individual’s nose or mouth. Exhaled material is typically collected before it becomes mixed with atmospheric air and is thus distinct from sampling indoor air from the patient room/environment. Articles that only tested aerosol samples from indoor air in COVID-19 patient environments, or that only tested swabs from any part of the respiratory tract were excluded, as were simulation studies, review articles, articles that are not original research studies, articles that describe study protocols only, and articles that were not published in English.

### Data extraction and evidence synthesis

Data were extracted onto pre-defined data extraction sheets by two independent reviewers in parallel and then were checked for inconsistencies. The data extracted included the period and country of study conduct, sample size and frequency, method of participant recruitment, method of confirming SARS-CoV-2, days since symptom onset, level of disease severity, SARS-CoV-2 variants tested, participant characteristics such as age, vaccination status, use of antiviral therapy or presence of immunosuppressive conditions, type of sample and method of collection, methods of laboratory testing and their outcome and trends with special attention to extracting information on viral load with the time since exposure. Upon data extraction, evidence was synthesized narratively and reported according to the PRISMA reporting standards [16].

### Assessment of bias

Eligible articles were then subjected to assessment of their internal validity and bias using a 10-item checklist which was developed using criteria appearing in both Joanna Briggs Institute [17] and National Heart, Lung, and Blood Institute [18] assessment tools. The assessment was carried out by two reviewers independently (AMHA, MW) and any inconsistencies were resolved through discussion involving a third reviewer (SGS). The questionnaire assessed studies on criteria involving sample selection, assessment and measurement of outcomes, and reporting of results. Possible options for each question were “yes,” “no,” “cannot determine” or “not applicable.” Based on the number of “yes” responses, each study was given a quality rating out of 10 as strong (8-10), moderate (4-7), or weak (0–3).

## Results

**Figure 1.**
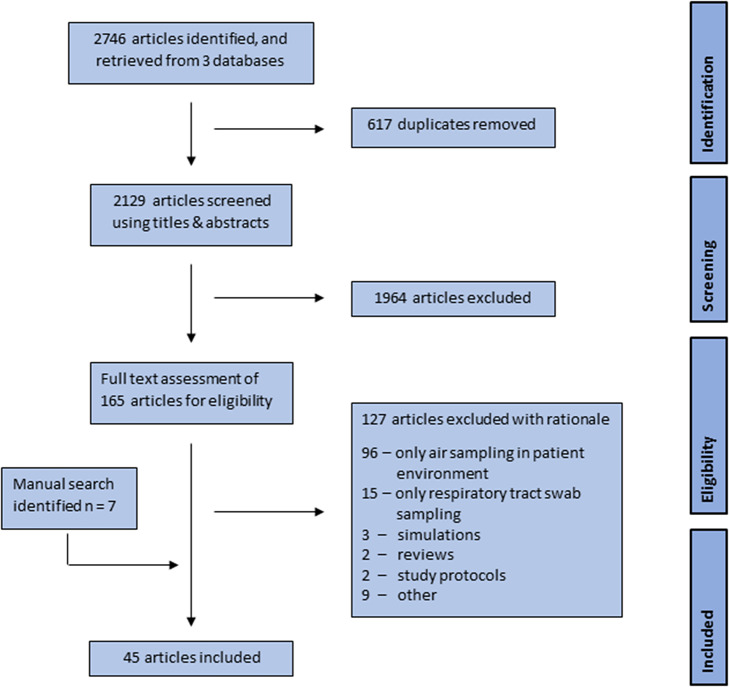
Flow diagram of the study selection process.

The initial search identified 2746 potential articles. Table 1 shows the number of articles identified in each of the databases and Figure 1 shows the study selection process. Six hundred and seventeen duplicates were removed before the title and abstract screening, which subsequently excluded an additional 1964 articles. Of the remaining 165 articles, full-text reviews excluded 127 which performed environmental air sampling in COVID-19 patient environments (*n =* 96), testing respiratory tract samples (*n* = 15), modelling or simulation studies (*n* = 3), reviews (*n* = 2), study protocols (*n* = 2) and other (*n* = 9). While exhaled breath is the source of viral material in the environment, environmental air samples do not directly represent the exhaled breath of an infected person, therefore these studies were excluded. A manual search of reference lists of eligible studies identified six records that were not identified via database search and one further study was included during the peer-review process as identified by the reviewer. The final sample consisted of 45 articles.

### Overall summary

Reviewed articles described studies conducted predominantly in China (*n* = 10) [19–28], the USA (*n* = 8) [29–36], and the UK (*n* = 4) [37–40]. Three studies each were reported from Canada [41–43], Germany [44–46], Sweden [47–49], and India [50–52]. France [53, 54], Japan [55, 56], and Singapore [57, 58] reported two studies each, and Belgium [59], Brazil [60], Ireland [61], South Korea [62], and the Netherlands [63] reported one study each. All studies were conducted between February 2020 and May 2022. Articles were published between 2020 and 2023, with over half (69%) published during 2022 and 2023. Most studies recruited hospitalized patients, including emergency department or intensive care unit admissions, with confirmed COVID-19 status. Other studies recruited health care workers (HCW) following routine testing [37, 48], patients from dialysis clinics [35, 36], close contacts of a case [47], and volunteers identified through advertising [39, 54], and a university health centre [31]. Study participants were recruited following SARS-CoV-2 positivity via testing of the nasopharyngeal swab (*n* = 26), throat swab (*n* = 6), saliva (*n* = 4), mid-turbinate swab (*n* = 3) or oropharyngeal swab (*n* = 3). One study each used sputum, endotracheal aspirates, and upper respiratory tract samples without being specific. Sample sizes ranged from 1 to 97 participants and 55.5% (*n =* 25/45) of studies used samples larger than 30. Serial (*n* = 15/45) and one-time sampling (*n* = 31/45) were reported while one study included one-time as well as repeated sampling of certain groups of study participants. Characteristics of reviewed articles are summarized in Table 2.

**Table 2.** Characteristics of reviewed studies

First author and publication year	Country	Sample and size	Range of days since symptom onset	Type/s of samples^a^	Sampling method	Sampling time/duration	Study quality
Adenaiye 2022 [29]	USA	Daily symptom report from local clinic, contact tracing *n* = 61	0–12	EB	Gesundheit-II (G-II) exhaled breath sampler	30 min	Moderate
Alsved 2023 [49]	Sweden	Case study *n* = 1	0–4	EB	Next generation impactor	30 min	Moderate
Alsved 2022 [47]	Sweden	Contact tracing *n* = 38	0–6	EB	BioSpot-VIVAS	30 min	Moderate
Coleman 2022 [57]	Singapore	Isolation patients *n* = 23	1–9	EB	Gesundheit-II (G-II) exhaled breath sampler	30 min	Moderate
Daniels 2021 [54]	France	Volunteers *n* = 14	NS	EBC	Mask-based polytetrafluoroethylene (PTFE) trap	5 min	Weak
de Man 2022 [63]	Netherlands	HCW *n =* 12 ICU patients *n* = 17	0–9^b^	Mask	Cutting out mask pieces	2.5 min	Moderate
Duan 2021 [30]	USA	Emergency department patients *n* = 70	NS	EBC	Bubbler	15 s	Moderate
Feng 2021 [19]	China	Hospitalized patients *n* = 15	EB: 13–28 EBC: 27–43	EB EBC	EBC: a sterile laboratory-made EBC collection system EB: exhaled aerosol collection system	30 min	Moderate
Gallichotte 2022 [31]	USA	University community *n* = 24 and *n* = 16 (two study groups)	NS	Mask	KN95 masks embedded with polyvinyl alcohol (PVA) strips	30 min to 3 h	Weak
Johnson 2022 [41]	Canada	Hospitalized patients *n* = 17	0–11	EB Mask Cough	BioSpot-VIVAS Mask rinse Cough bag/ cough on petri dish	Reading a 330-word English passage 25 coughs	Weak
Kim 2020 [62]	South Korea	Isolation patients *n* = 7	5–16	Mask	Swabbing surfaces of mask	5 coughs per mask	Moderate
Lai 2023 [32]	USA	PCR-confirmed cases from university *n* = 93	0–13	EB	Gesundheit-II (G-II) exhaled breath sampler	30 min	Moderate
Lane 2023 [33]	USA	Outpatients *n* = 60	0–20	EBC	A custom-made device	10 min	Moderate
Li 2022 [20]	China	Hospitalized patients *n* = 96	NS	EBC	BioScreen device	NS	Moderate
Li 2023 [21]	China	Hospitalized patients *n* = 78	NS	EBC	BioScreen device	NS	Weak
Li 2020 [22]	China	Hospitalized patients *n* = 1	NS	Mask	From the inside of the mask	NS	Weak
Lin 2022 [42]	Canada	*n* = 41 inpatients *n* = 34 community cases	1 - >90	Cough	Spontaneous or requested	3–5 min	Moderate
Lina 2022 [23]	China	Hospitalized patients *n* = 13	NS	EB	BioScreen device	5 min	Moderate
Lina 2023 [24]	China	Hospitalized patients *n* = 5	1–13^c^	EB	NS	NS	Weak
Ma 2021 [28]	China	Hospitalized patients *n* = 52	0–43	EBC	BioScreen device	5 min	Moderate
Malik 2021 [44]	Germany	Hospitalized patients *n* = 15	NS	EB	SensAbues	Exhale 20 times	Moderate
Malik 2023 [45]	Germany	Hospitalized patients *n* = 2	NS	EB	SensAbues	Exhale 20 times	Moderate
Mello 2022 [60]	Brazil	Hospitalized patients *n* = 45	NS	Mask	Cutting out mask pieces	2–3 h	Moderate
Nagle 2022 [53]	France	HCW *n* = 65	3–8.8 (IQR)	Mask	Inner surface of patients’ and external surface of HCWs’ mask	15 min	Strong
Nair 2023 [43]	Canada	Hospitalized patients *n* = 32 HCW *n* = 11	NS	Mask	Swabs of the inside of the surgical mask	NS	Weak
Pan 2023 [37]	UK	HCW *n* = 34	0–21	Mask	Polyvinyl-alcohol sampling strips placed in masks	1 h	Strong
Pfab 2023 [46]	Germany	Hospitalized patients *n* = 9	1–6	EB	Custom made device containing a filter paper	exhaled forcefully after a maximal inspiration	Moderate
Ryan 2020 [61]	Ireland	Hospitalized patients *n* = 40	2–20	EBC	RTube condenser	2 min	Weak
Sawano 2021 [55]	Japan	Hospitalized patients *n* = 50	3–7 (IQR)	EBC	R-tubeVent® device	5–7 min	Moderate
Sawano 2022 [56]	Japan	Hospitalized patients *n* = 41 Delta *n* = 45 wildtype	5–10 (IQR)	EBC	R-tubeVent® device	5–7 min	Moderate
Shrivastava 2023 [50]	India	Hospitalized patients Sample size not provided	NS	Cough	Cough on a petri dish	15 min	Weak
Smolinska 2021 [40]	UK	Hospitalized patients *n* = 48	1–14^b^	EB	Electrostatic filter attached to the face mask	30–60 min	Strong
Sriraman 2021 [51]	India	Hospitalized patients *n* = 31	3–8 (IQR)	EB	Gelatine membrane attached to the face mask	30 min	Moderate
Sriraman 2023 [52]	India	Hospitalized patients *n* = 92	3–5	EB	Gelatine membrane attached to the face mask	30 min	Strong
Stakenborg 2022 [59]	Belgium	*n* = 55 HCW *n* = 56 students n = 58 longitudinal trial	NS	EB	Tidal exhalations through the breath sampler	4, 2, and 1 min	Moderate
Tan 2023 [58]	Singapore	Hospitalized patients *n* = 47	0–15	EB	Gesundheit-II (G-II) exhaled breath sampler	30 min	Moderate
Verma 2022 [34]	USA	Hospitalized patients *n* = 97	4–5.25 (IQR)	EB	Gelatine membrane attached to the face mask	30 min	Moderate
Viklund 2022 [48]	Sweden	HCW *n* = 10 and *n* = 25 (two study groups)	0–11	EB	PExA (particles in exhaled air) BE (Breath Explor)	20 relaxed breaths	Moderate
Wang 2021 [35]	USA	Hemodialysis patients *n* = 14	0–38^b^	Mask	Swab from inner mask	3–4 h	Moderate
Wang 2023 [36]	USA	Hemodialysis patients *n* = 30	0–36^b^	Mask	Swab from inner mask	4 h	Moderate
Williams 2021 [38]	UK	Hospitalized patients *n* = 66	3–7	Mask	Polyvinyl -alcohol (PVA) strips attached to the mask	30 min	Strong
Zhang 2022 [25]	China	Unclear *n* = 85	0–17	EBC	BioScreen device	5 min	Moderate
Zheng 2022 [26]	China	Unclear *n* = 36	2–19	EBC	BioScreen device	5 min	Moderate
Zhou 2021 [27]	China	Patients recovering from COVID–19 *n* = 14	2–72	EBC	BioScreen II device	5 min	Moderate
Zhou 2023 [39]	UK	SARS-CoV–2 inoculated participants *n* = 36	0–14	Mask	NS	1 h	Moderate

EB, Exhaled breath; EBC, Exhaled breath condensate; HCW, Health Care Workers; NS, Not specified.

a Only relevant samples are listed;

b days since first positive test;

c days post hospitalization.

### Assessment of bias

The quality assessment of individual articles showed that a majority of articles (*n* = 31) were of moderate quality while others were weak (*n* = 9). Only a small number of articles (*n* = 5) were rated as strong. The information most commonly missing and which led to an assessment of moderate or lower quality was a clear description of participant enrolment and follow-up.

### Sample types and sampling methods

See Table 3 for a complete list of sampling methods. Four sample types were reported: 1) exhaled breath; 2) exhaled breath condensate; 3) face masks worn by patients; and 4) cough samples. The instruments used for exhaled breath sampling included the Gesundheit-II (G-II) exhaled breath sampler [29, 32, 57, 58], SensAbues [44, 45], BioSpot-VIVAS™ [41, 47] and custom-made devices [46, 59]. A BioScreen device was most commonly used for sampling exhaled breath condensate (*n* = 5). Masks were used to collect exhaled material by either swabbing the inner layer [22, 35, 36, 43, 53, 62], testing cut-out mask pieces [60, 63] or embedding sampling material (e.g. gelatine) within the mask [31, 34, 37, 38, 40, 51, 52]. Cough-only samples were tested in two articles [42, 50]. More than one sampling method was used in four articles [19, 41, 47, 48]. Two articles did not specify the sampling method. The sampling duration commonly reported was 30 min (*n* = 13), however, there was a wide range from 2 min to 4 h. Nine articles did not specify the length of sampling.

**Table 3.** Exhaled material types and methods of collection

Type of exhaled material	Method of collection	Mechanism of collection	Study
Exhaled breath	SensAbues (Sweden)	Filtration	[44, 45]
	PExA (particles in exhaled air) instrument (Sweden)	Cascade impactor	[48]
	Breath Explor (Sweden)	Impactor	
	Gesundheit-II (G-II) exhaled breath sampler (USA)	Slit impactor	[29, 32, 57, 58]
	BioSpot-VIVAS™ (USA)	Impactor	[41, 47]
	NIOSH sampler (USA)	Two-stage cyclone sampler	[19, 47]
	Custom made device	Filtration	[46, 59]
	Next Generation Impactor (Germany)	Cascade impactor	[49]
Exhaled breath condensate	BioScreen device (China)	Condensation	[20, [21], [23], [25]–28]
	Custom made device	Condensation	[19, 33]
	R-tubeVent™ (USA)	Condensation Designed specifically for intubated and mechanically ventilated patients	[55]
	Bubbler (USA)	Condensation	[30]
	RTube condenser (USA)	Cools exhaled breath, causing moisture to condense and collect	[56, 61]
	Polytetrafluoroethylene trap (USA)	Condensation	[54]
Face mask sampling	Swabs from inner mask	NA	[22, 35, 36, 43, 53, 62]
	Cut out pieces	NA	[60, 63]
Face mask embedded material	Gelatine		[34, 51, 52]
	Polyvinyl alcohol strips		[31, 37, 38]
	Electrostatic filter		[40]
Cough	Collected into polyethylene bags		[42]
	Petri dish		[50]

### PCR and shedding of viral RNA

All studies performed polymerase chain reaction (PCR) on samples of exhaled material to detect the presence of the virus. Many reported either cycle threshold (Ct) values or calculated a viral load or both. Gene targets for PCR were Nucleocapsids (N) (*n* = 28), Envelope (E) (*n* = 18), ORF1ab (*n* = 15), Spike (S) (*n* = 6), and RNA-dependent RNA Polymerase (RdRp) (*n* = 5). A majority of articles used a combination of these gene targets (*n* = 24). Detection limits of PCR assays were reported by 27% (12/45) of articles and they are summarized in Table 4. Only one article provided the limit of quantitation (“the lowest amount of the target in a sample that can be quantitatively determined with stated and acceptable precision and accuracy under stated experimental conditions” [64]) of their PCR assay which was 250 copies/sample [29]. Percentages of exhaled material positive for SARS-CoV-2 RNA ranged from 0 to 100%, with 18/45 articles having over 50% positivity.

**Table 4.** Detection limits of SARS-CoV-2 PCR assays reported (for 11 studies out of 44 included in this systematic review)

Limit of detection	Specification of the limit	Study
100 copies/μL	Diagnostic kit	[27, 28]
100 copies/mL	Diagnostic kit	[23, [24], 56]
500 copies/mL	Diagnostic kit	[49]
1,300 copies/mL	All sample types	[41]
10 copies/filter	Filters	[40]
100 copies/ filter	Gelatine filters	[34]
75 copies/sample	All sample types except saliva	[29, 32]
5 copies/reaction	All sample types	[62]

Changes in the viral load of exhaled material over time (longitudinal) were reported for different activities such as breathing, talking, and singing and are summarized in Table 5. Exhaled viral load during shouting was not reported as a separate activity. Cough-only sampling was reported, but its viral load changes over time were not assessed and this sampling method is discussed elsewhere in this review. The units used to report viral load varied between studies, making comparisons difficult. Eleven articles (24%) provided progressive viral load throughout infection for either one or more of these different activities. Not all articles that performed other activities (talking and singing) reported their respective values separately.

**Table 5.** Viral load (based on PCR) in exhaled material (exhaled breath/ exhaled breath condensate/ face mask) with time course of infection during breathing, talking, and singing (*n* = 10 longitudinal studies that reported these measures)

Days since symptom onset	Range of viral load shed during breathing	Range of viral load shed during talking	Range of viral load shed during singing
Day 0	ND copies/min [47] 6.25 x10^2^ copies/mL [49] Ct 33 - >40^a^ [59]	0–3.0x10^3^ copies/min [47] 7.0x10^3^ copies/mL [49]	0–7.7x10^3^ copies/min [47] 1.2x10^4^ copies/mL [49]
Day 1	ND copies/min [47] ND copies/mL [49] ND copies/patient [57] 1.7x10^5^ copies/min^b^ [23]	0–1.0x10^2^ copies/min [47] 2.5x10^3^ copies/mL [49] 8.0x10^1^ copies/patient [57]	0–3.8x10^2^copies/min [47] 6.0x10^3^ copies/mL [49] 7.1x10^2^ copies/patient [57]
Day 2	0–2.1x10^2^ copies/min [47] ND copies/mL [49] 0–1.4 x10^2^ copies/patient [57] 4.0x10^2^–3.2x10^2^ copies/mL [55] 1.0x10^4^–5.0x10^4^ copies/mL [56] Ct 25–33^a^ [59]	0–8.9x10^2^ copies/min [47] 5.0x10^2^ copies/mL [49] 0–7.3x10^2^ copies/patient [57]	0–1.1x10^3^ copies/min [47] 7.5x10^2^ copies/mL [49] 0–1.4x10^2^ copies/patient [57]
Day 3	0–2.3x10^1^ copies/min [47] ND copies/mL [49] 0–5.5x10^2^ copies/patient [57] 7.9x10^2^–2.0x10^3^ copies/mL [55] 1.3x10^2^–7.9x10^2^ copies/mL [56]	0–1.4x10^2^ copies/min [47] 2.0x10^3^ copies/mL [49] 0–4.3x10^3^ copies/patient [57]	0–1.9x10^3^ copies/min [47] 5.0x10^2^ copies/mL [49] 0–5.8x10^3^ copies/patient [57]
Day 4	ND copies/min [47] 0–6.4x10^1^ copies/patient [57] 1.0x10^3^ copies/mL [55] 5.0x10^2^–2.0x10^3^ copies/mL [56] Ct 26–34^a^ [59]	ND copies/min [47] 0–3.1x10^2^ copies/patient [57]	ND copies/min [47] 0–1.8x10^3^ copies/patient [57]
Day 0–4	0.5x10^1^–0.5x10^7^ copies/strip [38] 0–4.1x10^2^ copies/mL [25] 0–4.1x10^2^ copies/mL [25] 0–2.8x10^5^ copies/mL [26]		
Day 5	ND copies/min [47] 0–4.4x10^2^ copies/patient [57] 1.3x10^2^ copies/mL [55] 4.0x10^2^–1.3x10^3^ copies/mL [56]	ND copies/min [47] 0–2.4x10^3^ copies/patient [57]	ND copies/min [47] 0–1.2x10^3^ copies/patient [57]
Day 6	ND copies/min [47] ND copies/patient [57] 1.0x10^3^ copies/mL [55] 5.0x10^2^–7.9x10^2^ copies/mL [56] Ct 25–38^a^ [59]	ND copies/min [47] ND copies/patient [57]	ND copies/min [47] ND copies/patient [57]
Day 7	ND copies/patient [57] 1.0x10^2^ copies/mL [55] 4.0x10^2^–6.3x10^2^ copies/mL [56]	ND copies/patient [57]	ND copies/patient [57]
Day 8	ND copies/patient [57] 1.3x10^2^–1.3x10^3^ copies/mL [56] Ct 35 - >40^a^ [59]	ND copies/patient [57]	1.5x10^2^ copies/patient [57]
Day 5–8	0.5x10^1^–1.0x10^4^ copies/strip [38] 0–5.6x10^2^ copies/mL [25] 0–1.4x10^2^ copies/mL [25] 0–1.5x10^5^ copies/mL [26]		
Day 9	ND copies/patient [57] 3.2x10^4^ copies/mL [55] ND copies/mL [56]	ND copies/patient [57]	ND copies/patient [57]
Day 10	Ct 30 - >40^a^ [59]		
Day 9–12	1.0x10^1^ copies/strip [38] 0–6.5x10^1^ copies/mL [25] ND copies/mL [25] 0–4.5x10^4^ copies/mL [26]		
Day 15	3.2x10^4^ copies/mL [55]		
Day 16	3.2x10^3^ copies/mL [55]		
Day 13–16	ND copies/strip [38] ND copies/mL [25] ND copies/mL [25] 0–7.9x10^4^ copies/mL [26]		
Day 34	2.2x10^2^ copies/mL [19]		
Day 43	2.2x10^2^ copies/mL [19]		

ct, cycle threshold; ND, not detected.

a Days since first positive test;

b Days post hospitalization.

The range of viral RNA produced when talking and singing was generally higher than during breathing for a given study. Singing shed comparatively higher viral particles than talking, where reported. Overall, both activities (talking and singing) shed higher amounts of virus in exhaled material during the first 5 days of symptom onset and then went undetectable.

Three studies that reported viral load during each activity (breathing, talking, and singing) each day, either did not detect any or detected very low viral RNA while breathing on days 0 and 1 since symptom onset. They did, however, detect higher viral RNA for talking and singing on the same sampling days [47, 49, 57]. The highest loads of viral RNA shed in exhaled material while breathing were observed on days 2–5 since symptom onset [29, 47, 57]. Zheng et al. and Sawano et al. reported unusually high viral loads during the third week after symptom onset in fully vaccinated [26] and mechanically ventilated [55] patients. Two participants over the age of 60 and with severe symptoms produced detectable viral RNA in exhaled breath on days 34 and 43 [19]. Overall, beyond the first week after symptom onset, detection was generally low, although there was a wide variation in results.

Taken together, these 11 studies suggest that detectable RNA can commonly be recovered from exhaled particles during the first week of symptom onset with more vigorous activities such as singing being associated with higher detectable viral load compared to breathing alone.

### Viral culture and shedding of infectious virus

The presence of replication-competent virus in exhaled material was assessed by 22% (10/45) of articles. All of them performed viral culture, predominantly using Vero cells [29, 31, 32, 41, 42, 53, 57, 58], A549-ACE2 cells [29, 32, 58] or Caco2 cells [21]. Some used more than one cell type. One article did not mention the viral culture medium [39]. Viral culture was confirmed by observing for cytopathic effects [31, 53, 57, 58], plaque assay [39, 41, 42], or immunofluorescence staining and imaging [29].

Of the 10 articles assessing the presence of infectious virus, 80% detected viral growth from at least one sample of exhaled material. Table 6 provides a summary of these results. Two articles failed to isolate infectious viruses from exhaled material [57, 58], both of them used the Gesundheit-II exhaled breath sampler; however, a further two studies using this instrument did succeed in isolating live viruses [29, 32]. Three articles had study participants with mixed vaccination status [32, 53, 58], out of which one did not detect any infectious SARS-CoV-2 [58]. One study included unvaccinated and unexposed participants who were seronegative at enrolment [39]. These studies reported means of 4–5 days, and medians of 5–6 days and range from 0–9 up to 1- > 90 days since symptom onset. A specific pattern was not observed between the presence of infectious virus in exhaled material and days since symptom onset as this was variable (see Table 6 for more details).

**Table 6.** Detection of replication-competent virus from exhaled material in relation to days since symptom onset

Days since symptom onset			Infectious virus detected in [% (n)]					Study
Range	Median	Mean (SD)	Exhaled breath	Exhaled breath condensate	Mask	Cough only	Respiratory swab/saliva	
0–12		4.1 (2.5)	fine-aerosols: 3% (2/66) coarse-aerosols: negative					[29]
1–9			0					[57]
					4 (1/24)			[31]
0–11	5	5 (3.3)	0		0	25 (2/8)	53 (9/17)^a^	[41]
0–13		4 (2)	Delta 66.7 (2/3) Omicron 7 (2/29)					[32]
				BA.5 = 14.8 (4/27)				[21]
1 - >90						28 (10/36)	58 (23/40)^b^	[42]
IQR 3–8.8	6				6.25 (1/16)			[53]
0–15			0					[58]
0–14					50 (9/18)			[39]

IQR, Inter quartile range.

a Nasopharyngeal swab;

b saliva.

Zhou et al. presenting the first-in-human SARS-CoV-2 challenge study found a large variation in exhaled material (mask sampling) shedding and their infectiousness over the 14-day follow-up period [39]. Of the two high shedders (2 of the 18 participants who generated 86% of total airborne viral RNA), an infectious virus was recovered from the masks of only one participant. Other participants who had infectious virus recovered from their masks were identified as low shedders [39]. These replication-competent viruses were detected between 3 and 11 days after exposure [39]. Although study samples demonstrated a wide range of days since symptom onset (1- > 90), Lin et al. (2022) reported that infectious virus was typically detected in the first week after symptom onset [42]. Similarly, Nagle et al. also isolated their only positive infectious sample of exhaled material on day 5 after symptom onset [53]. Among different types of aerosols, Adenaiye et al. (2022) found that those with fine particles (≤5 μm) contained infectious virus (2 and 3 days post symptom onset) while those with coarse particles (>5 μm) did not [29]. Another similar study, however, did not have positive virus cultures from either type of particle [58]. Cough-only samples contained infectious material in 25–28% of samples tested [41, 42], also within the first week after symptom onset.

Only one article out of 10 that assessed replication-competent viruses provided quantitation data. The highest live viral load detected in a sputum cough sample was 1.3 × 10^6^ plaque-forming units (PFU)/mL (Ct N gene = 6.47), while unproductive cough resulted in viral loads ranging from 5 × 10^0^ to 1.9 × 10^3^ PFU/mL [42].

Taken together, these 10 studies suggest that replication-competent virus can be recovered from all sample types (exhaled breath, exhaled breath condensate, cough, and masks) and up to a week post-symptom onset.

### SARS-CoV-2 variants and exhaled material

A majority of articles (22/45) examined patients infected during mixed-variant periods while 20% (9/45) of studies were conducted before SARS-CoV-2 variants had emerged. Nine more articles (9/45) did not state the predominant variant involved. More than one SARS-CoV-2 variant type was sampled in 29% (13/45) of articles. Among these articles, patients infected with Alpha, Delta, and Omicron variants were assessed by eight articles each. Nine articles included other ancestral strains in the mix. Table 7 provides details on percentages of patients with each variant or subvariant included in these studies and their relevant findings.

**Table 7.** Percentage of participants with SARS-CoV-2 variants and respective findings relevant to exhaled material shedding

Alpha %(n)	Delta %(n)	Omicron %(n)	Other %(n)	Relevant findings	Study
8 (4/49)	–	–	Ancestral strains	Alpha variant associated with a 100-fold (95% CI, 16- to 650-fold) increase in coarse-aerosol and a 73-fold (95% CI, 15- to 350-fold) increase in fine-aerosol RNA shedding (after controlling for the effect of masks and numbers of coughs during sampling)	[29]
58 (22/38)	–	–	Ancestral strains	Not specified	[47]
18 (4/22)	4.5 (1/22)	–	Beta, Kappa 50 (11/22)	Not determined (due to small numbers of each variant)	[57]
4 (4/93)	3 (3/93)	31 (29/93)	Ancestral strains [61]	No significant differences observed between variant groups	[32]
–	11.2 (35/312)	20.8 (65/312)	Ancestral strains 62 (194/312)	No significant differences observed between variant groups	[33]
30 (29/96)	26 (25/96)	43.8 (42/96)	–	No significant differences observed between variant groups	[20]
–	–	BA.5 34.6 (27/79) BA.2 65.4 (51/78)	–	The average Breath Emission Rate of BA.5 patients was nearly 40 times higher than that of BA.2 patients	[21]
–	38.5 (5/13)	61.5 (8/13)	–	Omicron patients exhaled more virus particles than Delta patients at the first week of hospitalization	[23]
27.7 (18/65)	–	–	Ancestral strains 53.8 [35]	Not specified	[53]
33.3 (3/9)	22.2 (2/9)	–	Wild type 44.4 (4/9)	Not specified	[46]
–	47.7 (41/86)	–	Wild type 52.3 (45/86)	Delta patients exhaled significantly higher viral RNA load 2–8 days after symptom onset on a day-to-day basis, compared to wild type patients	[56]
8.2 (4/49)	10.2 (5/49)	42.9 (21/49)	Others 34.7 (17/49)	Omicron patients exhibited EB positivity that continued later into days since illness. Among the positive samples at ≥7 days, the positive detection rate of Omicron was higher than that of pre-Omicron variants	[58]
–	–	BA.2 60 (51/85) BA.1 40 (34/85)	–	BA.2 subvariant patients had higher EBC positive rate, compared with BA.1 subvariant patients	[25]
–	–	BA.2	–	The only patient in this case study had the highest concentration of SARS-CoV–2 RNA on the day of symptom onset and declined for each day thereafter (followed up to day 3 since symptom onset).	[49]

Adenaiye et al. (2022) found 18 times higher viral RNA shedding in patients infected with the Alpha variant than in patients infected with ancestral strains [29]. Infection with the Delta variant has also been shown to shed significantly higher viral RNA loads during the first week of symptom onset compared to wild-type infections [56]. Another study assessing five and six patients infected with Delta and Omicron variants, respectively, found a higher viral load in the exhaled breath in patients infected with Omicron during the first week of hospitalization [23]. The proportion of exhaled breath samples that tested positive by PCR later on (≥7 days since symptom onset) was higher among patients infected with Omicron variant viruses compared to pre-Omicron variants as reported by one study [58]. Among Omicron subvariants, patients infected with BA.5 had nearly a 40-fold higher breath emission rate compared to patients infected with BA.2 in one study [21], while another study found that patients infected with BA.2 subvariant had a higher proportion of positive exhaled material compared with BA.1-infected patients [25]. By contrast, three studies observed no significant differences in viral RNA shedding in exhaled material among the variants tested [20, 32, 33], while four articles either did not test or did not specify comparative assessments for the variants involved [46, 47, 53, 57].

Taken together, these studies suggest that more recent variants tended to be detected at higher viral loads than ancestral variants. However, variations in sampling methods and the timing and duration of sampling make it difficult to draw firm conclusions.

### Symptoms and exhaled material

Participants with a range of symptoms were assessed in reviewed articles, including asymptomatic patients, and patients with mild, moderate, and severe symptoms. Significantly higher viral shedding in exhaled material was reported from patients with more severe symptoms compared to those with mild, moderate, or no symptoms [33]. Patients who suffered a cough were found to be more likely to have higher viral shedding beyond the first week of symptoms onset [52].

## Discussion

This study systematically reviewed 45 publications that sampled exhaled material from SARS-CoV-2 infected people and tested for either viral RNA or live virus. The evidence comes from four continents (North and South America, Europe, and Asia) and diverse patient populations including inpatients, outpatients, volunteers, specific disease cohorts, and healthcare workers. The frequency of testing exhaled material increased as the pandemic progressed, with more studies published in 2022–2023 than in earlier years, possibly due to expanding knowledge on the sample type and increased acceptance that infectious SARS-CoV-2 could aerosolize. Reviewed evidence is highly variable in terms of sampling and testing methods as well as how results are presented. Therefore, these studies could not be combined to produce an overall parameter of the virus’ pathogenicity. This is clearly a missed opportunity and a deficit that future studies should address.

Although viral RNA was detected in exhaled material frequently over the first 14 days since symptom onset, the replication-competent virus can be found in aerosols within the first 8 days, similar to respiratory swab samples [65, 66]. Nevertheless, there was high variability in exhaled SARS-CoV-2 viral load by days since symptom onset and disease severity. This was also observed for other sample types [12]. One of the contributing factors for high variability of exhaled SARS-CoV-2 viral load could be the lack of standardization of breath sampling technique among studies. The sampling duration of reviewed studies varied from 15 s to 4 h. This could lead to the collection of samples containing differing amounts of virus, ultimately contributing to incompatible results among studies. Additionally, different sampling instruments were used to collect breath specimens. The quality of the sample collected will affect whether viral RNA can be detected. Most tests are designed with respiratory swab samples in mind, and exhaled materials have lower viral loads and therefore lower sensitivity compared with swab samples [67, 68]. Greater standardization of sample collection methods and testing protocols among studies would provide more robust summaries to inform disease modelling.

The gold standard diagnostic technique for SARS-CoV-2 viral RNA detection is the RT-PCR [69]. However, not all RT-PCR assays are the same, and sensitivity and specificity will vary among different PCR kits, in-house protocols, and based on the gene targets involved. For instance, assays targeting RdRP and E genes have been shown to have lower limits of detection, and thus higher sensitivity, compared to those that target the N gene [70], although the converse scenario has also been reported [71]. It is therefore crucial that studies report gene targets and assay detection limits to enable for informed interpretation of test results.

Viral shedding in exhaled material also differed by activity. Two studies compared talking, singing, and breathing and observed higher shedding of aerosol particles for singing and talking, compared to breathing alone [72, 73]. This is consistent with observed outbreaks and super-spreading at events involving group singing during the pandemic [74–76]. Notably, singing and talking generated exhales with detectable virus on days 0 and 1, while breathing did not. Nevertheless, none of these exhaled material groups showed consistent levels of SARS-CoV-2 above the limit of infectivity of 4.2 × 10^4^ copies/mL [77]. This has implications for public health policy and supports pandemic control recommendations to avoid participation in singing groups and indoor worship involving singing as well as attendance at loud venues that would necessitate shouting, such as bars, sporting events, and concerts [78, 79].

As well as infecting susceptible hosts via inhalation, aerosolized viruses can also be deposited on surfaces leading to fomite transmission. Regardless of the activity involved, mask positivity of COVID-19-positive patients and surface contamination were significantly linked [53], contributing to environmental contamination and higher infection risk. Contamination of surfaces such as common equipment, tables, and floors could lead to exposure and onward transmission, demonstrating that the implications of SARS-CoV-2 aerosolization are multifactorial.

Replication-competent SARS-CoV-2 has been recovered from all types of exhaled material via viral culture. Exhaled breath was the most commonly tested sample type (50% of the time [5/10]) and also the type of sample that most frequently resulted in zero detections (3/5). Exhaled breath condensate was the material least often attempted for infectiousness testing (1/10). Based on the limited studies available, the molecular diagnostic performance of exhaled breath condensate did not yield higher rates of detection [80]. Nevertheless, exhaled breath condensate has been identified to facilitate the diagnosis of COVID-19 in patients with high suspicion of infection in whom nasopharyngeal swab testing has returned negative results [61, 81]. Testing of face masks and cough specimens has shown considerable success in terms of detecting infectious SARS-CoV-2.

Coughing has been shown to result in exhalation of higher particle mass compared to other respiratory activities [82], which might also contain higher numbers of viral particles. Compared to cough samples, mask samples show a larger variability in viral load, as demonstrated by the changeability of the human housekeeping gene (18S rRNA). This variability may be a result of activities performed by the individual or alignment of the mask while sampling [39, 83, 84].

The evidence on the duration of infectious virus shedding in exhaled material throughout infection was scarce, constraining adequate reflection on this topic. The dearth of studies examining this outcome could be due to the need for Biosafety level three facilities, which dramatically increases the cost of experiments. Among those studies that did look at infectious viruses, many failed to report the length of illness of cases against the respective viral culture outcome. In the few studies where durations were clearly reported, viral culture commonly peaked 1 week after symptom onset and failed after this time. An infectious virus has also been detected before symptom onset [85]; however, among the studies reviewed here, only the experimental infection study [39] collected samples before symptom onset.

This review exclusively focused on exhaled SARS-CoV-2 from COVID-19-positive individuals throughout their illness. Although similar evidence on other common sample types such as upper respiratory tract swabs, stool samples and serum were readily available [12–15, 86], evidence specifically on exhaled material has not been synthesized previously. Our search was limited to three electronic database articles published in English, which may have restricted our search results.

Evidence reviewed here suggests that exposed individuals shed SARS-CoV-2 RNA in their exhale over the first 2 weeks since symptom onset, but that this exhaled material only contains infectious virus during the first week. However, the viral load in exhaled material is highly variable depending on the host and viral strain involved, as well as the type of activities performed by the individual. Symptom severity was an imperfect predictor of the shed viral load. This evidence is helpful in mitigating the risk of COVID-19 transmission in indoor spaces such as hospitals. For instance, a SARS-CoV-2 positive patient inactively lying in a coma state is likely to shed a low viral load and thus poses a low risk compared to a loud delirious patient in the emergency department who will pose a higher risk and would therefore require different infection control strategies. Summary parameters of viral shedding during different activities are useful for informing disease transmission models that in turn inform policy decision-making. When modeling the risk of nosocomial infection, the disease severity of the patient, and aerosol generation parameters of the host such as breathing, talking, coughing, immune status, use of masks, and virus variant involved should be considered in the model.

In conclusion, the current quantitative evidence on the viral load of exhaled material is scarce and variable, and a definitive duration and infectiousness of viral shedding via exhaled pathway is difficult to determine. There is a need for further experimental studies to assess exhaled material, their infectious status, and quantity over the course of infection. More consistent sampling methods and testing protocols are needed to enable greater comparison of results from different studies, to better understand the viral shedding by COVID-19 patients. Evidence from such studies will ultimately inform understanding of exposure risks associated with indoor environments, with implications for isolation and quarantine policies and regulations about indoor crowding and space management.

## Data Availability

The authors confirm that the data supporting the findings of this study are available within the article.
